# Social Frailty in Heart Failure: Concept, Impact and Preventive Strategies

**DOI:** 10.1111/jan.17037

**Published:** 2025-05-15

**Authors:** Miao Miao, Polly Wai‐Chi Li, Doris Sau‐Fung Yu

**Affiliations:** ^1^ School of Nursing, Li Ka Shing Faculty of Medicine The University of Hong Kong Hong Kong China

**Keywords:** health‐related quality of life, heart failure, self‐care, social frailty, social prescribing

## Abstract

**Aims:**

To explore the conceptualisation of social frailty and discuss its role in shaping the disease trajectory of heart failure. Based on the discussion, recommendations on how to prevent and manage social frailty in this clinical cohort are delineated.

**Design:**

A discursive paper.

**Methods:**

This paper searched two databases, PubMed and Google Scholar, for a narrative review of the literature related to social frailty and heart failure from 2008 to 2024.

**Findings:**

By integrating the conceptualisation of social frailty from different theoretical paradigms, social frailty is a multi‐domain construct that relies on a balance between the availability of environmental resources, social interactions and an individual's ability to maintain and acquire these resources to enhance their well‐being. Substantial evidence showed the prognostic impact of social frailty on patient‐reported, functional and clinical outcomes of patients with heart failure. The underlying mechanism is still under‐investigated, but heart failure‐related self‐care may mediate its impact. Based on this evidence, improving social frailty may rely on a diagnostic protocol to enhance the person‐centred care planning on ways to optimise the social resources to support complex self‐care.

**Conclusion:**

Social frailty poses a greater risk to health outcomes in patients with heart failure. Further research is needed to explore determinants and interventions for social frailty in this population.

**Implications:**

This paper increases the awareness of social frailty in heart failure patients and provides important insights on how to combat this social determinant of poor health outcomes among this clinical cohort. A dual‐purpose approach of improving social resources and self‐care behaviours may have great promise in reducing their social frailty, and this postulation will need to be investigated in future research.

**Patient or Public Contribution:**

There is no involvement of patients or the public in the design or writing of this discursive paper.

## 1 Introduction

As the concept of frailty evolves, related social issues have increasingly come to light, prompting the emergence and development of the concept of social frailty (Fujiwara et al. 2022). This concept emphasises the interplay between individual health and the broader societal context, highlighting its relevance in determining the health outcomes of clinical populations, particularly those experiencing physical debilitation (Nagai et al. 2020). Patients with heart failure often coexist with physical frailty (Pandey et al. 2019), which increases their susceptibility to social frailty. On the one hand, the high symptom burden has shrunk the ability of an individual to maintain their social network and engagement (Olano‐Lizarraga et al. 2022), which are regarded as crucial to preventing social frailty (Bunt et al. 2017). On the other hand, social frailty would compromise the support resources required by the patients to engage in complex self‐care for symptom management (Matsui et al. 2024). This vicious cycle, unique to heart failure patients, accelerates the loss of social resources and the deterioration of social functioning, far exceeding normal aging levels. Indeed, the prognostic impact of social frailty in heart failure has been reported (Long et al. 2025; Jujo et al. 2021; Watanabe et al. 2022). Nevertheless, heart failure management has provided the least consideration to the role of social frailty in shaping the disease trajectory. It is, therefore, imperative to critically review the evidence of social frailty in this clinical population so that an additional perspective from the social‐ecological paradigm can be integrated to optimise heart failure management.

## 2 Background

Heart failure is a complex clinical syndrome associated with structural and functional impairment of the heart that implies a hemodynamic disorder in which the filling or ejection of blood can't meet the body's needs (Heidenreich et al. 2022). It was considered an aging‐related cardiovascular condition (Gorodeski et al. 2018), with the mean age of this clinical cohort over 75 years (Norhammar et al. 2023). The prevalence was 1%–3% in the adult population (Ruiz‐García et al. 2023; Wang, Chai, et al. 2021), while it was much higher, from 6% to 14% in those aged over 60 years (Gorodeski et al. 2018). Heart failure was also confirmed to have a strong relationship with a poor prognosis, including a decline in quality of life, disability, depression, hospitalisation, high mortality within 1 year of admission and a low survival rate in 5 years (Dick and Epelman 2016; Ambrosy et al. 2014), leading to enormous caregiving and financial burdens on healthcare systems.

### 2.1 Frailty as a Co‐Existing Pathology to Complicate Heart Failure Trajectory

Frailty is a persistent and cumulative syndrome distinct from normal aging (Pandey et al. 2019). It is not only characterised by an imbalance between the organism's repair mechanisms and damage but also involves a decline in the ability to cope with internal and external stressors (Proietti and Cesari 2020; Chen et al. 2014). During the last decades, physical frailty has been the focus of attention. Due to the similarity in the etiological mechanism and manifestation, the mutual exacerbating effects between heart failure and frailty have been documented (Rich et al. 2016). Basically, the pro‐inflammatory state, impaired nutrient intake, chronic skeletal muscle abnormalities and high co‐morbidity burden induced by heart failure lead to pathological changes in muscle, weight loss and decreased physiologic reserve (Talha et al. 2023). All these changes indeed concur with the pathophysiologic mechanisms of frailty (Pandey et al. 2019; Talha et al. 2023). More importantly, the common symptom manifestation of heart failure and frailty, including fatigue, low endurance, low physical activity, malnutrition, anxiety and depression, would augment the debilitation to a great extent (Joseph and Rich 2017). A meta‐analysis found that the prevalence of prefrailty and frailty among heart failure patients was about 46% (*k* = 14, *N* = 50,156) and 40% (*k* = 20, *N* = 51,452), respectively (Marengoni et al. 2020). Other studies found that co‐existing frailty was a significant prognostic factor for more impaired function, poorer quality of life and higher mortality in this clinical cohort (Kleipool et al. 2018; Yang et al. 2018). It is noteworthy that the international clinical practice guideline of heart failure urges for proactive and effective strategies to enhance frailty management (Talha et al. 2023).

### 2.2 The Role of Domain‐Specific Frailty in Informing Care Mapping

For the sake of developing more sensitive strategies to tackle frailty, there is more recognition of the use of a domain‐specific approach to conceptualise frailty. By using this approach, frailty is regarded as a multidimensional concept that encompasses not only physiological aspects but also psychological and social dimensions to reflect the functioning decline in various domains. They can occur both independently and interconnectedly, collectively accelerating the process of frailty and leading to adverse health outcomes (Gobbens et al. 2010). Increasing evidence indicates the added values of this domain‐specific approach for sensitive case identification and effective care mapping (Tanaka et al. 2021). More specifically, in the context of heart failure, a meta‐analysis (*k* = 26 studies, *N* = 6896) indicated that the prevalence of frailty as identified by the studies that used multi‐domain approaches was higher than those that defined frailty in the sole physical aspect (Denfeld et al. 2017). This implies that heart failure patients who have preserved physical function may develop frailty in other functional domains. This finding was echoed by the FRAGILE‐HF cohort study, where the prevalence of physical, social and cognitive frailty varied from 37.1% to 66.4% (Matsue et al. 2020). More importantly, the inclusion of the number of frailty domains in the prognostic risk estimation was associated with a 22% net reclassification improvement for the combined rehospitalisation and mortality endpoint in 1 year (Matsue et al. 2020). Another study found that the prognostic impact of different domains of frailty varied among heart failure patients of different demography. For example, even though female heart failure patients are more susceptible to physical frailty, the male counterparts with this problem were at higher mortality and readmission risk (Aguilar‐Iglesias et al. 2024). Such strong associations between domain‐specific frailty and health outcomes imply that care mapping needs to be conducted in a domain‐specific fashion. This claim is supported by the European Society of Cardiology, which advocates using the domain‐specific approach to better address the dynamicity and synergism of different frailty subtypes along the heart failure trajectory (Vitale et al. 2019).

### 2.3 Social Frailty as the Least Addressed Aspect in Heart Failure Management

Social frailty is shaped by one's context and is represented by the extent an individual's social needs cannot be addressed due to the deprivation of resources (Bunt et al. 2017). This aspect of frailty is usually manifested as inadequate social engagement, ineffective social resources, lack of social support, or loneliness (Quach et al. 2021). Among the patients with heart failure, social frailty is closely related to the debilitating disease nature of heart failure, which compromises an individual's capability to maintain interaction with their social network (Wang, Fang, et al. 2021). The increasing care needs along the debilitating heart failure disease trajectory may also exceed the available social resources and result in inadequate social support (Quach et al. 2021). In fact, the FRAGILE‐HF cohort (*N* = 1240) found that social frailty has affected as many as two‐thirds of hospitalised heart failure patients aged 65 or older (Jujo et al. 2021).

However, social frailty has received relatively little attention in heart failure management. This oversight may arise from its unclear conceptualization of social frailty. The inadequate awareness of how social functional reserves influence the trajectory of heart failure may also explain the relatively lower priority given to the social determinants of health. Indeed, as a socially constructed concept, culture is known to play a crucial role in shaping social frailty through influencing the way an individual interacts with their social environment via the different beliefs, perceptions and attitudes (Fujiwara et al. 2022). The need for a more person‐centered and cultural‐sensitive assessment of social frailty would therefore further enhance support from a helping relationship. Addressing this clinical dilemma and research gap is crucial for developing proactive protocols to optimise social functional reserves and mitigate social frailty among heart failure patients.

## 3 Overview of the Issue

This discursive paper engaged with three key aspects of social frailty in relation to heart failure. First, it examined the conceptualisation of social frailty and its evolution along the HF disease trajectory. Second, this paper explored the prognostic impact of social frailty on heart failure and proposed potential underlying mechanisms. Based on the current state‐of‐the‐art on these, a vigorous discussion on the implications of tackling social frailty among this clinical cohort would be discussed.

## 4 Data Sources

This discursive paper provides a narrative synthesis of the literature on social frailty and heart failure. Two databases, PubMed and Google Scholar, were searched to retrieve literature related to social frailty and heart failure from 2008 to 2024. The following keywords were used for the search: social frailty, heart failure, concept, definition, prognostic impact, predictive and interventions. The focus of the search was to explore the development of social frailty, studies related to social frailty in patients with heart failure and potential interventions. We reviewed over 30 studies, including original articles, reviews and commentaries and engaged in a discussion of their viewpoints and conclusions. Among these, five articles focused on social frailty in heart failure patients, consisting of two cohort studies (Jujo et al. 2021; Watanabe et al. 2022) and three cross‐sectional studies (Uchmanowicz et al. 2015; Li et al. 2022; Wang et al. 2023). Due to the limited research on older adults with heart failure, we discussed evidence related to social frailty in community‐dwelling seniors and considered the characteristics and specific needs of heart failure patients. Additionally, we referenced studies on components of social frailty to gather further evidence, allowing us to deduce the underlying mechanism and potential intervention strategies for social frailty in older adults with heart failure.

## 5 Findings

### 5.1 The Conceptualization of Social Frailty

The concept of social frailty has evolved over the past two decades, but no consensus has yet been reached. Andrew et al. first proposed an operational definition of social vulnerability based on the deficit accumulation approach (Andrew et al. 2008). The terminology of ‘social frailty’ was first introduced by Gobbens et al. (Gobbens et al. 2010) when he proposed the Integral Conceptual Model of Frailty (ICMF) to define frailty as a multidimensional phenomenon. Following such development, Bunt et al. (2017) conducted a scoping review based on the Social Production Function (SPF) theory to further advance the understanding of the conceptual domains of social frailty, and the findings are coherent with a systematic review aimed at operationalising these domains (Bessa et al. 2018). Below is the integration of all these foundation works for the sake of thoroughly elucidating the concept of social frailty.

Andrew et al. proposed ‘social vulnerability’ in a deficit accumulation approach of frailty (Andrew et al. 2008; Andrew 2015). The core concept of this approach is that the cumulative deficits of an individual would increase one's vulnerability to adverse health outcomes. On the other hand, those with more preserved resources or functions are more likely to resist or mitigate the threats to health. On this basis, social vulnerability is defined as the accumulation of multiple socially related deficits that render individuals more susceptible to health‐related adverse events and social challenges (Mah 2023). Besides, social vulnerability would manifest at different levels of one's social spheres, including individuals, family and friends, peer groups, institutions and the wider society, and the accumulated effects across these sectors offer a means to quantify this undesirable state. Contrary to this concept is ‘social reserve’, which was proposed by Andrew et al. as the potential resource and support stemming from one's social environment to buffer the threats from stressors such as aging and disease (Andrew 2015). In other words, social vulnerability is preventable and reversible. A clear delineation of the scope of social reserve may offer important insights on how to resist, delay and reverse social frailty.

The term ‘social frailty’ as first introduced in the integral conceptual model of frailty proposed by Gobbens and his colleagues in 2010 (Gobbens et al. 2010). After that, Bunt et al. (2017) integrated the research evidence relating to the components of social frailty and proposed its definition based on the Social Production Function Theory (SPF). Central to the SPF theoretical framework is the hierarchy of well‐being through which one's physical and social well‐being can only be achieved if the basic needs have been addressed (Ormel 2002). Social frailty is, therefore, conceptualised as a high risk of persistent shortages of social resources to meet the basic social demands for affection, behavioural confirmation and social status throughout the life span (Bunt et al. 2017). As compared with the initial concept of social vulnerability proposed by Andrew, social frailty based on the SPF provides more specification on the resources needed to prevent the depriving situation, including general resources, social activities and social support. Besides, Bunt et al. (2017) also proposed self‐management as a unique influencing factor other than resources to determine social frailty. Self‐management refers to the critical ability of an individual to proactively mobilise, manage and maintain the resources in the process of achieving well‐being (Steverink and Lindenberg 2008). Defining social frailty using the SPF theoretical paradigm therefore provides perspectives to determine the level of social frailty by balancing an individual's needs against one's ability to mobilise the available resources.

Based on the above conceptualization of social frailty, this construct reflects a process of continuous loss of socially important resources, whereby the person's interaction with the external environment is reduced, and ultimately leads to a deficit in fulfilling the need for wellness. Indeed, social frailty can be regarded as ‘multi‐dimensional’, as this problem resulted from the cumulative loss of a different resource. A systematic review of social frailty measurements revealed that the major components of social frailty encompass social isolation, loneliness, social networks, social support and social engagement (Bessa et al. 2018). This finding echoes the conceptualization of social frailty proposed by Andrew et al. (2008) and Bunt et al. (2017) that social frailty is related to the complex interplay of both external and internal social resources. Whereas the external resource refers to the concrete tangible resources and social interactional components, the internal resource focuses on one's perceptual aspect of social contentedness and one's ability to secure the social connection.

Integrating existing concepts enhances our understanding of the resources related to social frailty. Importantly, this concept does not merely reflect the quantity or quality of resources an individual possesses. Instead, it focuses on identifying individuals who experience an imbalance between resources and needs. Recognising this imbalance will provide insights for effective interventions. In other words, optimising internal and external resources to maintain the stability of this balance will be a key focus in preventing social frailty. At the same time, supplementing critical resources or alleviating demands to promote the restoration of balance will be crucial in reversing social frailty.

### 5.2 The Prognostic Implications of Social Frailty in Heart Failure

The prognostic impact of social frailty on the health and wellness of older adults has been widely documented (Li et al. 2023). Although evidence regarding heart failure patients is still limited, both direct and indirect evidence suggests that social frailty negatively impacts health outcomes in this population. This phenomenon may be related to the decline in self‐care associated with heart failure.

In heart failure patients, a cohort study of 1240 individuals found that after adjusting for demographic and clinical risk factors, social frailty was associated with a 1.3 times increased risk of all‐cause mortality and heart failure readmission (Jujo et al. 2021). Another retrospective cohort study (*N* = 310) reported consistent findings on the prognostic risk of this composite outcome (adjusted HR = 2.01, 95% CI = 1.07–3.78) (Watanabe et al. 2022).

In fact, different domains of social frailty also affect the health outcomes of patients with heart failure. Friedmann et al. (2014) found that low social support predicted worsening depressive symptoms in heart failure patients, which was highly associated with a significant decline in physical functioning over 6 months. Social isolation also plays a part, with research indicating its negative impact on increased emergency service use, 90‐day hospital readmission and overall healthcare utilisation (Polikandrioti 2022; Heidari Gorji et al. 2019). Another study demonstrated the prognostic impact of other social frailty elements, including living alone, financial burden and poor social networks among heart failure patients (Leigh‐Hunt et al. 2017). Social frailty is, therefore, a more integrative social determinant of health among this clinical population, and the negative health impact of each domain may be synergistic rather than additive. Elucidating the underlying mechanism of how social frailty worsens heart failure health outcomes is imperative to inform the corresponding care advancement.

### 5.3 The Mechanism Explaining the Prognostic Impact of Social Frailty

There is a dearth of evidence to explain the prognostic impact of social frailty among patients with heart failure. Studies examining how social frailty affects the way patients manage the disease (Uchmanowicz et al. 2015; Li et al. 2022) suggest that self‐care may be a crucial mediator in explaining the associated prognostic impact.

Heart failure self‐care is defined as a proactive decision‐making process to engage in health‐promoting behaviours and more complex disease management strategies to maintain physiological stability and health (Riegel et al. 2012). This concept covers three important aspects, including self‐care maintenance, symptom perception and self‐care management (Riegel et al. 2012). In the context of heart failure, effective self‐care requires patients to have good compliance with the treatment regime and lifestyle modification (i.e., self‐care maintenance), be sensitive to and able to interpret the early symptom manifestation (i.e., symptom perception) and make sound decisions to take proactive action to manage the symptoms and early disease deterioration (i.e., self‐care management). Substantial research evidence indeed reported the protective effect of self‐care on patient‐reported and clinical outcomes of patients with heart failure (Moser et al. 2012; Liljeroos et al. 2020). International clinical practice guidelines published by the Heart Failure Association of the European Society of Cardiology in 2020 therefore stated that promoting self‐care is a priority in the health management for this clinical cohort (Jaarsma et al. 2021).

However, the complexity of heart failure‐related self‐care implies the important role of the social context in shaping its effectiveness. Social frailty may indicate the lack of social resources and support to enable the patients to engage in complex disease management. Previous studies consistently identified a positive association between social frailty and poorer self‐care among both Western and Chinese heart failure patients (Uchmanowicz et al. 2015; Li et al. 2022). More specifically, social isolation was found to hinder access to healthcare (Tanaka et al. 2021), which resulted in delayed care seeking during disease exacerbation. On the other hand, good social support was consistently found to bolster the confidence, motivation and adherence to self‐care behaviours among this clinical cohort (Graven and Grant 2014). Together with a range of social barriers to self‐care, including financial burden, social isolation, poor social support, low health literacy and transport limitations delineated in the 2022 American Heart Association/American College of Cardiology/Heart Failure Society of America guideline (Heidenreich et al. 2022), tackling social frailty may require effective intervention to enhance the social context of the patients in these dimensions. Buffering strategies to support the self‐care of heart failure patients with social frailty are warranted.

## 6 Discussion

Although there are no investigations on how to improve social frailty in heart failure patients, the conceptualisation of social frailty and the potential mechanism to explain its health impact in this clinical cohort may provide insights into strategies to reverse social frailty. As a multi‐dimensional construct that relies on the balance between the availability of social resources from multiple aspects and an individual's need for such resources to maintain wellness, a person‐centred approach to improve social frailty is crucial. This would, in turn, imply the need for a sensitive assessment of social frailty for patients with heart failure. As for optimising their social frailty, efforts can be placed on optimising the different domains of social frailty, especially those highly relevant to support their self‐care.

### 6.1 The Disease‐Specific Domains for the Assessment of Social Frailty

Accurate assessment of social frailty is a prerequisite to ensure patient‐centered strategies to address this clinical issue. As previous research points to the role of self‐care in explaining the prognostic impact of social frailty in heart failure patients, an effective assessment of social frailty may need to capture the dimensions that may hinder the patient's capacity to manage self‐care. Up to our knowledge, there is no disease‐specific measure for social frailty. Although there are several tools to measure social frailty, none of them consider the unique social needs of heart failure patients and their preferences and methods for accessing social resources. The more common measurements include Makizako's screening tool for social frailty, the Social Frailty Scale, the HALFT (Help, Participation, Loneliness, Financial, Talk) scale and the Social Frailty Index (SFI) (Montayre et al. 2024). These questionnaires mainly cover social economic status (e.g., living conditions, financial difficulties, educational level, social networks, etc.) and proactive social behaviours (e.g., going out, visiting friends, participating in social and social activities, helping others, etc.), but overlook the bidirectional flow characteristics of social resources. For heart failure patients who are more likely to have physical limitations due to the symptom burden, their social engagement may rely on methods such as online chatting or visits by family members. Strong social support can help buffer the negative impact of activity limitations on social needs (Wang et al. 2023). This dynamic compensation of social resources can be understood through the holistic perspective of the ecological framework. Resources are fluid and interwoven, circulating among various levels, including individual, interpersonal, organisational, community and public policy. When the accumulated deficits of resources across the ecological framework exceed the capacity for self‐compensation, the entire support system may collapse, limiting health management behaviours and exacerbating the negative impact on health (Miao and Yu 2024).

However, the conventional social frailty measures, which do not consider the preferences and compensatory forms of social resources and connections (Montayre et al. 2024), may lead to an overestimation of the problem for this clinical cohort. On the other hand, the more important aspects of social interaction and resources for heart failure patients may be related to those that can support effective self‐care maintenance, symptom interpretation and self‐care management. Disease‐specific items that can capture the corresponding social context would be important to ensure the contextual relevancy of the measurement.

Another aspect of social frailty assessment that is specific for heart failure patients is about the optimal schedule for the corresponding health monitoring. For these patients, the loss of internal and external resources is inevitable as they age and the occurrence of symptoms undoubtedly exacerbates the progression of social frailty. Heart failure patients would typically undergo a graduated trajectory of functional deterioration interspersed with repeated episodes of cardiac decompensation. Their need for social support resources and their ability to engage in the social context would vary along the disease trajectory. Additionally, life events can further exacerbate or alleviate social frailty. Research has shown that negative events, such as a spouse being injured or ill, significantly increase the risk of social frailty, while positive events, such as making new friends, can reduce this risk and even reverse social frailty (Makino et al. 2024). Consequently, when heart failure patients face an acute decline in internal resources or external resources, and these losses cannot be compensated, they are at a heightened risk of social frailty. Timely identification of the needs of this population is crucial for preventing or reversing social frailty. Therefore, it is essential to develop disease‐specific monitoring plans that consider the impact of the disease on the process of social frailty and adequately assess the resilience of the individual's resource reserves to inform proactive and personalised care planning.

### 6.2 Strategies for Optimising Social Resources for Heart Failure Patients

Social frailty is a multi‐dimensional concept for which previous studies have identified the more important roles of social support, social roles and social interaction needs (i.e., living alone is an independent prognostic indicator) in association with the prognostic impact. Strategies to improve social frailty should therefore focus on addressing these three aspects.

For social support, the intervention can be designed in a way to optimise the self‐care of this clinical cohort. A systematic review (*k* = 11, *N* = 2098) found that heart failure patients who received verbal encouragement from family members and significant others on self‐care (i.e., emotional support), guidance from healthcare providers (i.e., informational support) and tangible resources such as preparation for medications, low‐sodium foods, etc. (i.e., instrumental support) reported greater motivation to engage in disease‐related health behaviours (Babygeetha and Devineni 2024). Another systematic review (*k* = 8, *N* = 1623) summarised interventions for social dimensions in patients with heart failure, suggesting that strategies such as increased knowledge of the disease, psychosocial support, communication strategies and motivational enhancement have the potential to improve social support and self‐care (Olano‐Lizarraga et al. 2023).

Regarding the social role, an effective social dynamic with family members is critical to allow the role adaptation of heart failure patients in coping with the debilitating disease process. More recently, more attention has been directed to the importance of social dynamics and relationships within the patients‐caregiver dyads in the context of heart failure. The Theory of Dyadic Illness Management proposed by Lyons and Lee (2018) provides an important framework to guide the development of strategies to optimise the interactions within the heart failure patients and caregiver dyads. Central to this theory is that the care dyads need to act as interdependent teams to achieve congruent illness perception. Mutual collaboration in disease management, with an emphasis on addressing the needs of both parties, is essential to optimising the health outcomes of both. In this process, the reciprocal relationship and collaborative social dynamic may introduce better‐perceived role functioning of heart failure patients. A recent systematic review (*k* = 48, *N* = 9171 dyads), indeed, concludes the importance of dyadic dynamic optimisation in optimising the self‐care and health outcomes of this clinical cohort (Yu et al. 2024).

As for addressing social interaction needs for heart failure patients, consideration needs to be given to how activity intolerance has restricted their social engagement within a more shrunken physical environment. Tackling social frailty in this aspect may require optimisation of home‐based social activities. Care robots, online peer support groups and virtual social platforms may be possible means to address their social interaction needs. A randomised controlled trial examined the effects of robotic pets on social frailty in community‐dwelling older adults, demonstrating that interactions with robotic pets have the potential to improve social frailty and a dose–response relationship was noted (Pollak et al. 2022). Information communication technologies (ICTs), such as telemedicine, e‐health and m‐health, can be considered (Azizi et al. 2024). These technologies can promote the accessibility and timeliness of social resources for heart failure patients by sharing patient monitoring data with healthcare professionals, thereby assisting in disease management, early identification of deterioration and provision of professional advice (Yu et al. 2024). Yet, a parallel intervention to empower the patients' engagement in these platforms is needed and a more person‐centred approach to address their digital literacy and care preferences appears to be imperative to determine the interventional effectiveness.

## 7 Conclusion

Although social frailty is a developing concept, it has been recognised that the loss of various types of social resources and mobilising capacity increases an individual's vulnerability and leads to a decline in health. Older adults with heart failure have a higher risk of social frailty compared to the general population and trigger faster disease progression and poorer health outcomes. Therefore, it is urgent to study social frailty in people with heart failure to identify determinants and interventions. Moving forward, the effectiveness of interventions with the dual goals of supplementing social resources and improving self‐care in preventing and reversing social frailty needs to be validated by more research. There is also a need to develop and design innovative ways of patient‐friendly social resource support for heart failure patients. More importantly, the findings can support the construction and implementation of public policies to provide social protection from a macro perspective and ultimately combat social frailty.

## Author Contributions

Conceptualisation: Doris Sau‐Fung Yu, Miao Miao. Involved in drafting the manuscript: Miao Miao. Involved in revising it critically for important intellectual content: Doris Sau‐Fung Yu, Polly Wai‐Chi Li. Given final approval of the version to be published. Each author should have participated sufficiently in the work to take public responsibility for appropriate portions of the content: Doris Sau‐Fung Yu. Agreed to be accountable for all aspects of the work in ensuring that questions related to the accuracy or integrity of any part of the work are appropriately investigated and resolved: Doris Sau‐Fung Yu, Miao Miao, Polly Wai‐Chi Li.

## Conflicts of Interest

The authors declare no conflicts of interest.

## Data Availability

Data sharing not applicable to this article as no datasets were generated or analysed during the current study.
